# Evaluation of the effect of dental anxiety on vital signs in the order of third molar extraction

**DOI:** 10.1186/s12903-024-04596-w

**Published:** 2024-07-25

**Authors:** Elif Esra Özmen, İsmail Taşdemir

**Affiliations:** 1 Ahmet Keleşoğlu Faculty of Dentistry, Department of Oral and Maxillofacial Surgery, Karamanoğlu Mehmetbey University, Karaman, 70100 Turkey; 2 Ahmet Keleşoğlu Faculty of Dentistry, Department of Periodontology Dentistry, Karamanoğlu Mehmetbey University, Karaman, Turkey

**Keywords:** Dental anxieties, Dental phobia, Tooth extractions, Vital sign, Systolic pressure, Diastolic pressure

## Abstract

**Background:**

Dental anxiety is a prevalent concern affecting patients undergoing various dental procedures, particularly surgical interventions. Understanding the impact of patients’ anxiety levels on their physiological responses during dental surgeries, such as third molar impaction surgery, is crucial for optimizing patient care and outcomes. Therefore, this study aimed to investigate the effect of patients’ anxiety levels on vital signs during third molar teeth impaction surgery.

**Methods:**

A cross-sectional study was conducted, including 45 randomly selected, healthy patients. Demographic information was recorded after obtaining consent from the patients prior to surgical intervention. Preoperative anxiety levels were determined using the Modified Dental Anxiety Scale (MDAS). Pupil measurements were taken from the patients before surgery, at 10 min after the surgery began, and at 10 min after the surgery ended. Systolic (SBP) and diastolic (DBP) blood pressure, pulse rate, temperature, and haemoglobin oxygen saturation (SpO_2_) values were recorded.

**Results:**

The MDAS test results were statistically significantly higher in women compared to men (*p* < 0.001). Positive correlations were observed between MDAS score and both preoperative pulse rate (*r* = 0.344, *p* = 0.021) and SpO2 level during the operation (*r* = 0.462, *p* = 0.001). However, no significant correlations were found between MDAS and DBP (*p* = 0.575), SBP (*p* = 0.176), fever (*p* = 0.238), or pupil diameter (*p* = 0.338).

**Conclusions:**

Third molar impaction surgery induces anxiety in adult patients 20 years and older. Vital sign monitoring provides information about the patient’s emotional state, both before and during the procedure. Since anxiety causes changes in vital signs during dental procedures, it is important to follow these findings to have an idea about the general condition of the patients.

## Background

Dental anxiety can be defined as fear and apprehension that arise from dental treatment [1, 2]. Anxiety can manifest itself in different age groups, sexes, and social classes for various reasons [3, 4]. Dental fear, on the other hand, is described as a combination of several anxieties, such as fear of pain, fear of losing a tooth, and/or the thought of treatment as unpleasant or painful [2]. Traumatic dental experiences, personal characteristics, sex, age, and education level influence a patient’s level of dental anxiety [5]. Psychological and environmental factors also play a role in its development [6].

Anxiety involves both psychological and physiological responses, including headaches, dizziness, irregular heartbeats, difficulty breathing, sweating, increased blood pressure and pulse rate, trembling, tachycardia, arrhythmia, and facial flushing or pallor [7]. In addition, physiological dilation of the pupils has been reported in stressful situations [8].

Third molar surgery at the age of 20 years, a procedure commonly performed in the Turkish population, is often associated with high levels of anxiety and discomfort [9]. Dental anxiety can occur even when making an appointment or waiting in the waiting room. It is commonly associated with the sight of a syringe, the sensation created by injections, and the use of a handpiece [10–12].

Increased preoperative anxiety levels are associated with an increased need for intraoperative anaesthetic, higher perioperative pain perception, and an increased need for analgesics after surgery. Moreover, this psychosocial instability can trigger hemodynamic changes that may result in unwanted cardiovascular effects during surgery [13, 14].

While previous studies have explored the impact of anxiety on physiological parameters such as pulse and blood pressure during dental surgeries, fewer have investigated additional indicators such as temperature and haemoglobin oxygen saturation (SpO2) [13, 14]. To our knowledge, no study has examined changes in pupil diameter as a marker of anxiety during third molar extraction surgery.

Hence, this study aims to evaluate vital signs, including pulse, blood pressure, temperature, and SpO2 levels, along with pupil diameter changes, in individuals undergoing third molar extraction surgery. By examining these physiological markers, we seek to deepen our understanding of the relationship between dental anxiety and vital signs during surgical procedures, providing insights that may inform patient care practices and improve overall treatment outcomes. This study hypothesizes that individuals with higher levels of dental anxiety will exhibit greater fluctuations in vital signs, including pulse, blood pressure, temperature, and SpO2 levels, during third molar extraction surgery compared to those with lower anxiety levels. Additionally, we hypothesize that pupil diameter will increase in individuals experiencing heightened anxiety during the procedure.

## Materials and methods

### Study design and sample

This study was designed as a cross-sectional prospective investigation and received approval from the Karamanoğlu Mehmetbey University Clinical Research Ethics Committee (decision no. 06–2023/07). The research was conducted between August 2023 and December 2023 with the participation of 45 volunteers diagnosed with dental problems. Prior to their participation, all participants were required to provide informed consent. The study adhered rigorously to the principles outlined in the Declaration of Helsinki throughout all stages of the research process. We have meticulously reported the findings of this study in accordance with the Strengthening the Reporting of Observational Studies in Epidemiology (STROBE) statement to ensure the highest quality of reporting.

The study included 45 healthy volunteers (age, 18–60 years) who applied for impacted third molar extraction at the Department of Oral and Maxillofacial Surgery, Faculty of Dentistry, Karamanoğlu Mehmetbey University Ahmet Keleşoğlu and agreed to participate in the study.

Patients with serious neurological, cardiac, respiratory system disorders, uncontrolled diabetes, haematological diseases, those who had undergone head and neck radiotherapy, those using bisphosphonate-derived drugs, pregnant individuals, and those with alcohol or drug addiction were excluded.

### Sample size

In the pre-study phase, a total of 10 individuals were included in the study. The G*Power software package (Version 3.0.10, Franz Faul, Kiel University, Germany) was used to calculate the required sample size for this study. For a repeated measures analysis of variance (ANOVA) with three time points (preoperative, intraoperative, postoperative), an effect size of 0.20, an alpha level of 0.05, and a power of 0.80 were considered. According to these parameters, a sample size of approximately 42 participants was deemed necessary. However, to account for potential dropouts and missing data, we aimed to recruit 45 participants. This sample size is sufficient to detect medium to large effect sizes in the context of dental anxiety and physiological response studies, providing robust and reliable results.

### Surgical procedure

**Fig. 1:**
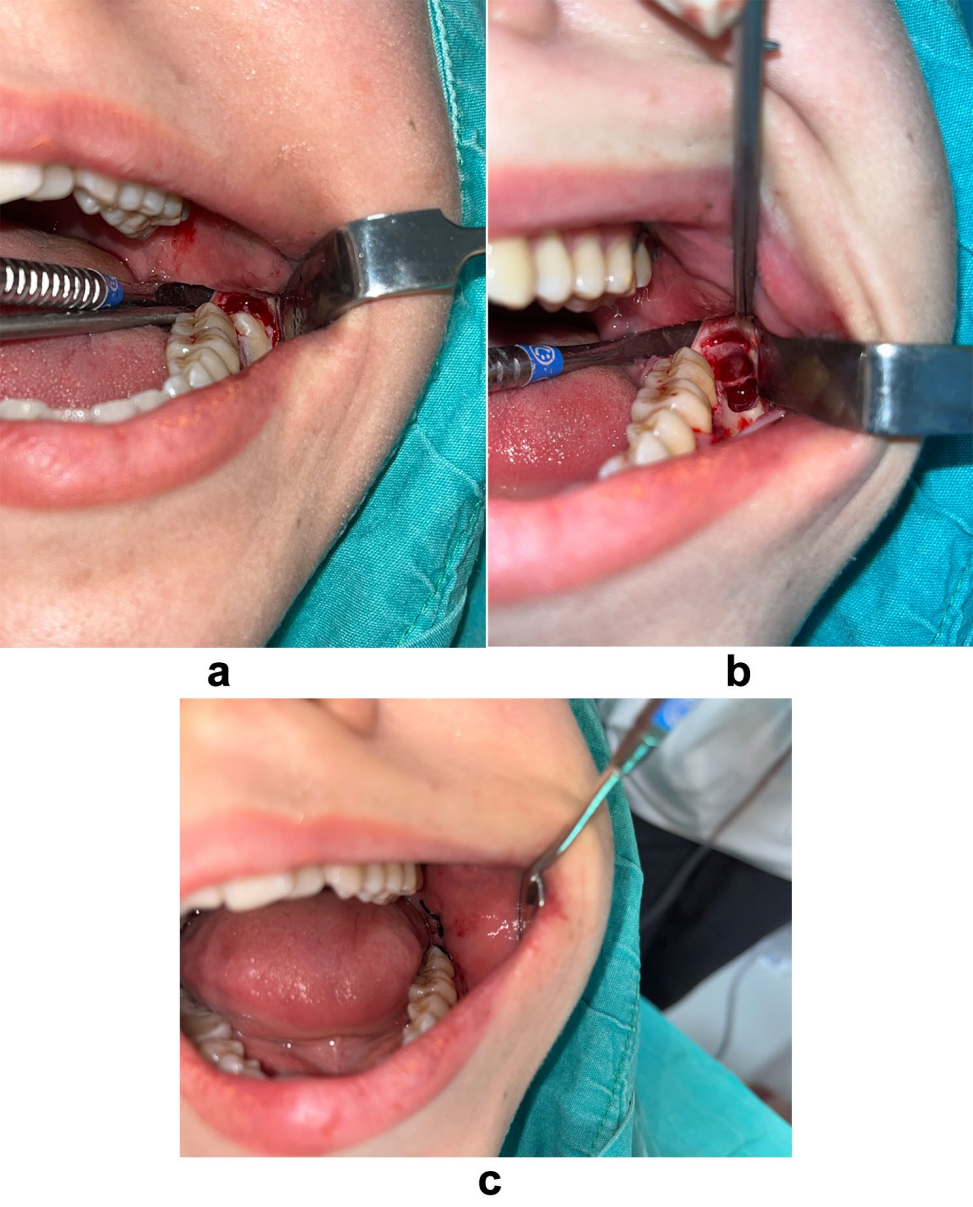
(**a**) Surgical image after raising a full-thickness flap. (**b**) Surgical image after tooth extraction (**c**) Surgical image after suturing

The study participants underwent a standardised procedure for third molar extraction. Perioperative procedures and surgeries were performed consistently according to the guidelines set by the Department of Oral and Maxillofacial Surgery at Ahmet Keleşoğlu Faculty of Dentistry. After covering the surgical field, a preparation containing articaine and epinephrine (1:100,000) was applied to block the inferior alveolar nerve and lingual nerve. Infiltration anaesthesia was administered to induce numbness in the buccal mucosa. Five minutes after the procedure, the numbness level resulting from infiltrative and mandibular anaesthesia was checked. An incision extending vertically to the distal half of the cervical edge of the second molar from the retromolar region was made, and a full-thickness flap was raised with a periosteal elevator. Osteotomy was performed under rinsing with physiological saline, and effective curettage was applied after the extraction of the impacted tooth. If the patient complained of intermittent pain or discomfort during the procedure, limited additional anaesthetic solution was administered, and the procedure was paused until the patient’s discomfort subsided. After surgery, routine postoperative instructions, a wound care plan, haemostasis, and usage of prescribed medications were explained to the patient. The application of ice was recommended to all patients to reduce postoperative swelling. Surgical photographs were taken with a Canon EOS 7D camera (Canon, Tokyo, Japan) (Fig. 1a, b and c).

### Outcome measures

#### Modified dental anxiety scale (MDAS)

The MDAS was utilised to assess preoperative dental anxiety. MDAS consists of five scores with answers to each question ranging from ‘not anxious’ to ‘extremely anxious’. The total score ranges from 5 to 25, and the score increases with the severity of dental anxiety. Scores ≥ 19 indicate severe anxiety. It has reasonable psychometric properties, low instrumental effects, and can be integrated into daily dental practice [15].

#### Beck depression score (BDS)

The Beck Depression Score (BDS) was used to measure the severity of depression in patients. The BDS consists of 21 multiple-choice questions, with each question scored on a scale from 0 to 3. The total score can range from 0 to 63, with higher scores indicating more severe depressive symptoms. The BDS has been extensively validated and is widely used in both clinical and research settings. In this study, it was employed to assess whether depressive symptoms correlated with dental anxiety and physiological responses to third molar extraction. Participants completed the BDS preoperatively to evaluate their baseline level of depression [16].

#### Vital signs

Vital sign evaluation included monitoring of blood pressure, pulse rate, SpO_2_ level, fever, and pupil diameter measurements. Measurements were taken before surgery, 10 min after the surgery began, and 10 min after the surgery ended [17].

### Blood pressure

Blood pressure measurements were taken with the patient in a seated position in the dental unit using an Erka classic spiral sphygmomanometer (Aneroid Perfect model) on the left arm twice, with a 15 s interval between readings. The average values of systolic blood pressure (SBP) and diastolic blood pressure (DBP) were recorded in millimetres of mercury (mmHg) [17].

### Pulse rate and haemoglobin oxygen saturation (SpO2)

Pulse and SpO_2_ values were measured in a seated position using a G Life Pulse Oximeter (Model: YK-81 A; Germany) on sweaty fingers after cleaning the probe, with results recorded in beats per minute and percentage (%) [17].

### Fever

Body temperature was measured to monitor any changes indicating stress or infection. Measurements were taken using a digital infrared tympanic thermometer (Braun ThermoScan 7 IRT6520, Germany) in the patient’s right ear. This method provides accurate and quick readings. The thermometer was calibrated and cleaned between uses to ensure accuracy and prevent cross-contamination. The recorded temperature was noted in degrees Celsius (°C) [17].

### Pupil/iris ratio

**Fig. 2:**
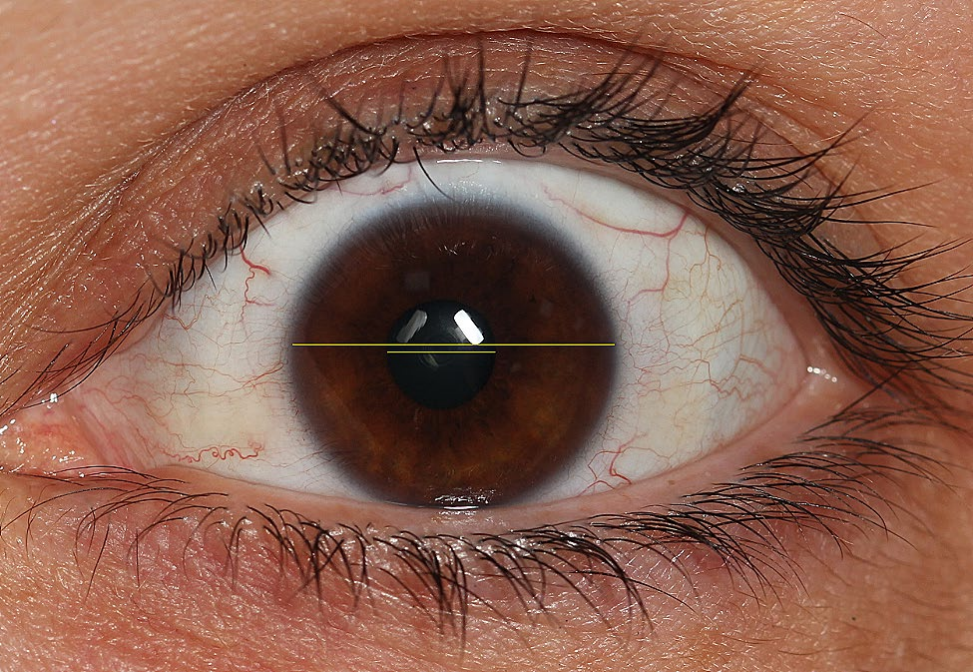
Study’s method of evaluating the pupil/iris ratio, which is a parameter we use to evaluate the change in the eye during application

Photographs of iris diameter and eye pupil diameter were taken with a Canon EOS 7D camera (Canon, Tokyo, Japan), and dimensions were obtained using Digimizer image analysis software (MedCalc Software, Ostend, Belgium) [18]. Iris diameter and eye pupil diameter were measured using a measuring tape, and their ratio was determined (Fig. 2).

### Statistical analysis

SPSS data analysis program was used to examine the data in this study. In all groups, Skewness and Kurtosis values ​​were determined between − 1 and + 1. Repeated Repeat ANOVA test was used to compare repeated measures of normally distributed data groups. As a result of the repeated anova test, it was checked whether Mauchly’s Sphericity Assumption was met. In the results where Mauchly’s Assumption of Sphericity is met, *p* values ​​are greater than 0.05, indicating that there is no difference between the data in that group. In groups where differences emerged, multiple intragroup comparisons were made with the Bonferroni test.

​The correlation between BDS and MDAS data was measured with Pearson correlation analysis. Again, using Pearson correlation analysis, the correlation of all measurement groups, BDS and MDAS data was examined.

## Results

The Table 1 presents the mean, standard deviation, minimum, and maximum values of various vital measurements taken before (1), during (2), and after (3) the operation. These measurements include SBP, DBP, pulse rate, haemoglobin oxygen saturation, body temperature, MDAS scores, and pupil/iris ratio.

**Table 1:** Vital measurements before, during, and after surgery

Measurements	Mean	Sd	Min	Max
SBP1 (mmHg)	117.33	13.718	90	150
SBP2 (mmHg)	115.67	15.797	80	150
SBP3 (mmHg)	112.89	13.077	90	150
DBP1 (mmHg)	76.22	6.839	60	90
DBP2 (mmHg)	74.00	8.090	60	90
DBP3 (mmHg)	73.33	8.790	60	90
Pulse 1 (BPM)	96.56	12.330	70	124
Pulse 2 (BPM)	97.18	15.627	64	130
Pulse 3 (BPM)	93.27	15.296	61	130
Saturation 1 (%)	95.64	2.414	90	99
Saturation 2 (%)	95.22	2.325	90	99
Saturation 3 (%)	96.36	2.013	91	101
Fever 1 (°C)	36.598	0.1631	36.2	36.9
Fever 2 (°C)	36.476	0.3016	35.7	37.1
Fever 3 (°C)	36.544	0.1829	36.2	36.9
MDAS	10.51	4.813	5	22
P/I 1	0.2882	0.04951	0.19	0.37
P/I 2	0.2744	0.04620	0.19	0.38
P/I 3	0.2762	0.04499	0.21	0.38

Mean, standard deviation, minimum and maximum values of vital measurement findings before (1), during (2) and after (3) the operation are given. (Sd: Standard deviation, min: Minimum, Max: Maximum, SBP: systolic blood pressure, DBP: diastolic blood pressure, MDAS: modified dental anxiety scale, P/I: Pupil/iris ratio, mmHg: Millimetre of mercury, BPM: Beats per minute, %: Percentage, °C: Celsius)

The Table 2 presents the dental anxiety of the participants according to gender. These results indicate that there are significant differences in dental anxiety levels between male and female participants, with females exhibiting higher levels of anxiety as measured by both the BDS and MDAS scales (*p* < 0.001).

**Table 2:** Gender differences in Dental anxiety levels

		*n*	Mean	Sd	t	df	*p*
BDS	Male	21	5.71	2.901	-3.687	31.061	< 0.001
	Female	24	11.63	7.216			
MDAS	Male	21	7.81	1.861	-4.084	29.232	< 0.001
	Female	24	12.54	5.316			

Independent sample t test was used to detect differences between male and female in dental anxiety. Independent sample t test, *p* < 0.05* (n: Number of participants, Sd: Standard deviation, BDS: Back depression scale, MDAS: Modified dental anxiety scale)

The Table 3 presents the changes in various vital signs before, during, and after the operation. The factors examined include SBP, DBP, pulse rate, saturation, fever, and pupil/iris ratio. These results suggest that while there were no significant changes in systolic blood pressure (*p* = 0.88), pulse rate (*p* = 0.68), or pupil diameter (*p* = 0.081) during the operation, significant changes were observed in diastolic blood pressure (*p* = 0.044), saturation (*p* = 0.014), and fever levels (*p* = 0.022), indicating alterations in these vital signs in response to the surgical procedure.

**Table 3:** Effects of Surgical Operation on Vital signs

		sum of squares	df	mean squares	F	*p*
SBP (mmHg)		453.704	2	226.852	2.497	0.88
	**Mistake**	7996.296	88	90.867		
DBP (mmHg)		205.926	2	102.963	3.243	0.044*
	**Mistake**	2794.074	88	31.751		
Pulse (BPM)		397.511	1.642	242.064	2.969	0.68
	**Mistake**	5891.156	72.256	81.532		
Saturation (%)		29.526	2	14.763	4.493	0.014*
	**Mistake**	298.141	88	3.286		
Fever (°C)		0.338	1.634	0.207	4.370	0.022*
	**Mistake**	3.402	71.896	0.047		
P/I		0.005	1.696	0.003	2.723	0.081
	**Mistake**	0.082	74.642	0.001		

Repeated measures ANOVA test results. Repeated ANOVA test, *p < 0.05 significant (SBP: Systolic blood pressure, DBP Diastolic blood pressure, P/I: Pupil/iris ratio, mmHg: Millimetre of mercury, BPM: Beats per minute, %: Percentage, °C: Celsius)

The Table 4 presents the correlation analysis between MDAS scores and various pre-operative measurements, including BDS, SBP1, DBP1, pulse rate (Pulse 1), saturation, fever, and pupil/iris ratio (P/I 1). Age shows a weak positive correlation with BDS (*r* = 0.205, *p* = 0.177) and a weak negative correlation with MDAS (*r* = -0.215, *p* = 0.157), although these correlations are not statistically significant (*p* > 0.05). MDAS scores exhibit a significant positive correlation with saturation (*r* = 0.344, *p* = 0.021) and a weak positive correlation with fever (*r* = 0.151, *p* = 0.338). SBP1 shows a moderate positive correlation with MDAS scores (*r* = 0.462, *p* = 0.001) and a weak positive correlation with saturation (*r* = 0.205, *p* = 0.176). Pulse 1 demonstrates a significant positive correlation with saturation (*r* = 0.353, *p* = 0.017). Fever 1 exhibits a weak positive correlation with saturation (*r* = 0.180, *p* = 0.238). These results suggest that pre-operative MDAS scores are positively associated with saturation levels and systolic blood pressure, indicating a potential link between dental anxiety and physiological responses such as increased heart rate and decreased oxygen saturation levels. However, the correlations with other pre-operative measurements are weak or non-significant.

**Table 4:** Associations between preoperative variables and Dental anxiety

		BDS	MDAS	SBP1	DBP1	Pulse 1	Saturation 1	Fever 1	*P*/I 1
Age	**r**	0.205	− 0.215	0.106	− 0.086	− 0.137	− 0.306*	− 0.193	0.150
	**p**	0.177	0.157	0.488	0.575	0.369	0.041*	0.204	0.324
MDAS	**r**			0.025	− 0.009	0.344*	0.151	0.146	− 0.099
	**p**			0.873	0.953	0.021*	0.322	0.338	0.517
SBP1	**r**				0.205	0.462*	− 0.119	0.404*	0.006
	**p**				0.176	0.001*	0.438	0.006*	0.968
DBP1	**r**					0.034	0.096	0.155	0.114
	**p**					0.827	0.531	0.308	0.456
Pulse 1	**r**						− 0.016	0.353*	0.082
	**p**						0.916	0.017*	0.594
Saturation 1	**r**							0.183	− 0.028
	**p**							0.230	0.854
Fever 1	**r**								0.180
	**p**								0.238

Correlation analysis of MDAS and pre-operative measurements. Pearson correlation, *p < 0.05 significant. (BDS: Back depression scale, MDAS: Modified dental anxiety scale, SBP: Systolic blood pressure, DBP: Diastolic blood pressure, P/I: Pupil/iris ratio)

The Table 5 presents the correlation analysis between MDAS scores and measurements taken during the operation, including SBP2, DBP2, pulse rate (Pulse 2), saturation, fever, and pupil diameter (P/I 2). Age demonstrates weak correlations with SBP2 (*r* = -0.050, *p* = 0.742), DBP2 (*r* = -0.118, *p* = 0.441), Pulse 2 (*r* = -0.063, *p* = 0.683), Saturation 2 (*r* = -0.247, *p* = 0.102), Fever 2 (*r* = -0.011, *p* = 0.940), and pupil/iris ratio 2 (*r* = 0.116, *p* = 0.447), but none of these correlations are statistically significant (*p* > 0.05). MDAS scores exhibit a weak negative correlation with SBP2 (*r* = -0.109, *p* = 0.475), weak positive correlations with DBP2 (*r* = 0.110, *p* = 0.473) and Pulse 2 (*r* = 0.276, *p* = 0.067), and a significant positive correlation with Saturation 2 (*r* = 0.369, *p* = 0.012). However, there are no significant correlations between MDAS scores and Fever 2 (*r* = 0.095, *p* = 0.535) or pupil/iris ratio 2 (*r* = 0.216, *p* = 0.153). SBP2 demonstrates a significant positive correlation with DBP2 (*r* = 0.397, *p* = 0.007) and Saturation 2 (*r* = 0.256, *p* = 0.089), while Pulse 2 shows a significant positive correlation with Saturation 2 (*r* = 0.221, *p* = 0.145). However, there are no significant correlations between SBP2 and Pulse 2, or DBP2 and any other measured variables. Saturation 2 exhibits a weak negative correlation with pupil/iris ratio 2 (*r* = -0.111, *p* = 0.468), but this correlation is not statistically significant (*p* > 0.05). Fever 2 demonstrates no significant correlations with any other measured variables. These results suggest that MDAS scores during the operation are significantly correlated with changes in saturation levels, indicating a potential influence of dental anxiety on physiological responses such as decreased oxygen saturation. However, correlations with other measurements during the operation are generally weak or non-significant.

**Table 5:** Associations between intraoperative variables and Dental anxiety

		SBP2	DBP2	Pulse 2	Saturation2	Fever 2	*P*/I.2
Age	**r**	− 0.050	− 0.118	− 0.063	− 0.247	− 0.011	0.116
	**p**	0.742	0.441	0.683	0.102	0.940	0.447
MDAS	**r**	− 0.109	0.110	0.276	0.369*	0.095	0.216
	**p**	0.475	0.473	0.067	0.012*	0.535	0.153
SBP2	**r**		0.397*	0.256	− 0.054	0.373*	− 0.024
	**p**		0.007*	0.089	0.726	0.012*	0.874
DBP2	**r**			0.104	− 0.024	0.069	0.182
	**p**			0.497	0.875	0.653	0.230
Pulse 2	**r**				− 0.031	0.221	− 0.041
	**p**				0.842	0.145	0.787
Saturation 2	**r**					− 0.102	− 0.111
	**p**					0.504	0.468
Fever 2	**r**						− 0.034
	**p**						0.822

Correlation analysis of MDAS and measurements made during the operation. Pearson correlation, *p < 0.05 significant (MDAS: Modified dental anxiety scale, SBP: Systolic blood pressure, DBP: Diastolic blood pressure, P/I: Pupil/iris ratio)

The Table 6 presents the correlation analysis between MDAS scores and postoperative measurements, including SBP3, DBP3, pulse rate (Pulse 3), saturation, fever, and pupil diameter (P/I 3). Age demonstrates weak correlations with SBP3 (*r* = -0.051, *p* = 0.740), DBP3 (*r* = -0.063, *p* = 0.683), Pulse 3 (*r* = -0.172, *p* = 0.257), Saturation 3 (*r* = -0.198, *p* = 0.193), Fever 3 (*r* = -0.060, *p* = 0.695), and pupil/iris ratio 3 (*r* = 0.174, *p* = 0.253). However, none of these correlations are statistically significant (*p* > 0.05). MDAS scores exhibit a very weak positive correlation with SBP3 (*r* = 0.005, *p* = 0.975), a moderate negative correlation with DBP3 (*r* = -0.245, *p* = 0.104), and a weak positive correlation with Pulse 3 (*r* = 0.202, *p* = 0.183). However, there are no statistically significant correlations between MDAS scores and Saturation 3 (*r* = 0.197, *p* = 0.195), Fever 3 (*r* = 0.162, *p* = 0.287), or pupil/iris ratio 3 (*r* = 0.094, *p* = 0.538). SBP3 shows a significant positive correlation with DBP3 (*r* = 0.349, *p* = 0.019), as well as a significant positive correlation with Saturation 3 (*r* = 0.409, *p* = 0.005). However, there are no significant correlations between SBP3 and any other measured variables. DBP3 demonstrates no significant correlations with any other measured variables. Pulse 3 exhibits a weak positive correlation with Fever 3 (*r* = 0.142, *p* = 0.352), but this correlation is not statistically significant (*p* > 0.05). Saturation 3 demonstrates a weak negative correlation with Fever 3 (*r* = -0.058, *p* = 0.707), but this correlation is not statistically significant (*p* > 0.05). Fever 3 demonstrates no significant correlations with any other measured variables. These results suggest that postoperative MDAS scores are not significantly correlated with most postoperative measurements, indicating that dental anxiety may not have a substantial impact on these physiological responses following the surgical procedure. However, there are significant positive correlations between SBP3 and DBP3, as well as SBP3 and Saturation 3, suggesting potential associations between these variables.

**Table 6:** Associations between postoperative variables and Dental anxiety

		SBP3	DBP3	Pulse 3	Saturation 3	Fever 3	*P*/I.3
Age	**r**	− 0.051	− 0.063	− 0.172	− 0.198	− 0.060	0.174
	**p**	0.740	0.683	0.257	0.193	0.695	0.253
MDAS	**r**	0.005	− 0.245	0.202	0.197	0.162	0.094
	**p**	0.975	0.104	0.183	0.195	0.287	0.538
SBP 3	**r**		0.349*	0.409*	− 0.040	0.287	0.146
	**p**		0.019*	0.005*	0.795	0.056	0.337
DBP 3	**r**			− 0.183	0.047	0.174	0.136
	**p**			0.230	0.759	0.252	0.373
Pulse 3	**r**				0.038	0.142	− 0.075
	**p**				0.803	0.352	0.622
Saturation 3	**r**					− 0.161	− 0.058
	**p**					0.290	0.707
Fever 3	**r**						0.165
	**p**						0.280

Correlation analysis of MDAS and postoperative measurements. Pearson correlation, *p < 0.05 significant (MDAS: Modified dental anxiety scale, SBP: Systolic blood pressure, DBP: Diastolic blood pressure, P/I: Pupil/iris ratio)

## Discussion

We examined the impact of the dental anxiety level of patients undergoing third molar surgery on vital signs, including SBP, DBP, pulse, respiration, fever, and pupil/iris ratio.

Despite numerous innovations in dentistry, anxiety related to dental procedures still persists, with some studies suggesting that it even surpasses the fear of heights [19]. The type of dental treatment influences anxiety, and extensive research has been conducted on this topic. While some researchers claim that root canal treatment causes the highest anxiety, others suggest that tooth extraction induces the highest due to patients’ perceptions of injections and disruption of body integrity [20–23]. Several studies have identified factors affecting anxiety and fear associated with tooth extraction [24, 25]. In this study, we aimed to investigate the effects of anxiety related to lower third molar surgery on vital signs.

To this end, we used the MDAS. Many studies have emphasised the appropriateness and reliability of the MDAS for evaluating anxiety in the Turkish population [26, 27]. Physiological responses, including blood pressure, pulse, skin temperature, and respiratory rate, are among the physiological measures used to assess anxiety. Heart rate is considered a useful measure during dental procedures [28]. Physiological responses are known indicators of anxiety and pain levels [13], in the form of increased blood pressure, acceleration of the heart rate and breathing, and muscle tension as coping mechanisms in response to stress [29, 30]. In Smolen et al., positive relationships between anxiety level during colonoscopy and pulse and blood pressure were reported in a high-anxiety group [31]. Similarly, in this study, positive significant correlations were found between MDAS score and preoperative and intraoperative pulse measurements.

In previous studies, the highest pulse rate was reported to occur when the patient first sat in the chair and during incision and flap elevation, while it decreased significantly after the operation [32]. Similarly, we observed positive correlations between MDAS score and preoperative and intraoperative pulse readings. Arda et al. compared saturation values before, during, and after the procedure in patients undergoing magnetic resonance imaging (MRI) [33]. There were significant differences between preoperative and postoperative saturation values and saturation values during the procedure, reportedly due to a possible increase in anxiety levels of patients during MRI. Twari et al. studied children requiring pulpectomy procedures and found that the highest pulse oximeter values were obtained before starting the procedure in a high-anxiety group. They suggested that this may have been due to anxiety-related hyperventilation [34]. In this study, a significantly positive correlation was observed between the SpO_2_ measurement taken during surgery and the MDAS score. This was attributed to increased anxiety levels during the operation.

In this study, when evaluating pupil-iris diameter percentage ratios, no significant difference was observed between MDAS and pupil diameter. The ratio of pupil diameter to iris diameter was utilized in pupil diameter measurements, as the width of the iris remained constant. In the conducted studies, the iris diameter in facial photographs has been indicated as a reliable and repeatable measurement method using individual oculometric references [35]. Although pupil diameter tends to change in anxiety situations, this study did not reveal a significant difference. Despite their advantages, pupil size measurements are infrequently employed in arousal detection due to interference effects associated with lighting conditions and cognitive effort [18]. It is important to note that changes in pupil diameter are influenced by various factors beyond anxiety. Changes in pupil diameter can be employed for various stimuli, including images and sounds [8].

In addition, when evaluating SBP and DBP values at three time points independently of anxiety, no significant differences were found in SBP for the three measurements, whereas the preoperative value DBP was significantly higher than the intraoperative and postoperative measurements. Changes in SBP and DBP may be observed in patients before and during clinical dental procedures. These changes tend to be associated with stress related to pain, fear, and anxiety during surgery, as well as individual factors such as age, hypertension, traumatic experiences in dental treatment, psychological response, poor eating habits, sedentary lifestyle, body mass index, and tobacco use [36]. After an operation, blood pressure readings generally return to a normal level. This is due to the highest level of anxiety being observed before and during the operation; i.e., anxiety levels decrease to a minimum after the procedure is over [14].

The absence of a significant difference in SBP at the different measurement times may be related to the overall low anxiety levels of the study participants. It is also essential to consider that anxiety levels can vary among individuals, and a more diverse sample may provide a broader understanding of the relationship between anxiety and vital signs.

Another factor that can affect dental anxiety is a patient’s sex. Some studies have shown that women have higher anxiety levels than men [37, 38]. In this study, we also observed that the MDAS score was higher for women [39]. This may suggest that men are generally more emotionally stable than women [40]. In addition, men are more likely to conceal their emotions. In general, higher dental anxiety scores among women may indicate that women express their emotions more freely than men [41].

It is also true that there is a connection between the perception of pain and dental anxiety. In the literature, there are many studies show that dental anxiety leads to an increase in the perception of pain during procedures [42, 43].

In the study, anesthesia with adrenaline was applied and adrenaline was deemed to have no effect on vital signs. This decision was influenced by the fact that Ogunlewe et al. achieved fertility during tooth extractions performed under anesthesia with and without adrenaline, which did not have any effect on vital signs [44].

This study explored the impact of dental anxiety on vital signs during third molar surgery, revealing significant correlations between anxiety levels and changes in physiological parameters such as pulse and SpO2. These findings are consistent with previous studies that have shown the influence of anxiety on physiological responses during dental procedures [14, 28, 31]. Recent studies have also focused on the post-operative sequelae of third molar avulsion, particularly facial oedema and pain. For instance, a systematic review and meta-analysis examined whether kinesio taping reduces pain, swelling, and trismus after mandibular third molar surgery, suggesting potential benefits [45]. Another review investigated the use of photobiomodulation to reduce postoperative pain, edema, and trismus, showing promising results [46]. Additionally, a randomized controlled trial evaluated the effect of a pre-operative single dose of prednisone on three-dimensional facial swelling, providing evidence for its effectiveness [47]. Moreover, a systematic review explored whether listening to music reduces anxiety and pain during third molar surgery, indicating positive outcomes [48]. These studies highlight the multifaceted nature of managing post-operative sequelae and suggest that combining anxiety reduction techniques with other interventions such as piezosurgery and laser medication may enhance patient outcomes [49]. Future research could further explore these combined approaches to provide a comprehensive strategy for managing both anxiety and post-operative symptoms in dental surgery.

While this study provides valuable insights into the relationship between dental anxiety and vital signs during third molar extraction surgery, it is essential to acknowledge several limitations that may have influenced the results and interpretation. Firstly, while the findings of this study suggest a correlation between MDAS scores and pupil diameter, it’s essential to recognize that pupil diameter changes can be influenced by various emotional and cognitive factors beyond anxiety alone. Therefore, the generalizability of results may be limited by the complexity of emotional responses and the potential interplay of multiple feelings affecting pupil diameter. Moreover, the study focused exclusively on the Turkish population, which may restrict the applicability of the results to other demographic groups with different cultural backgrounds and healthcare systems. Additionally, the measurement methods employed, while standard clinical techniques, could introduce variability due to differences in devices and operator techniques. The subjective nature of anxiety assessment using the MDAS may also introduce bias into the results. Furthermore, being a single-center study, the findings might not fully represent the variability of experiences and outcomes observed across different clinical settings. The cross-sectional design of the study limits the ability to establish causality between dental anxiety and changes in vital signs, warranting further longitudinal investigations. Lastly, variations in anaesthesia and pain management techniques were not accounted for in this study, potentially influencing patients’ anxiety levels and vital signs during surgery. Despite these limitations, this study contributes to the existing literature on dental anxiety and vital signs, highlighting the importance of considering psychological factors in dental care. Future research addressing these limitations could provide a more comprehensive understanding of the complex relationship between anxiety and physiological responses during dental procedures. However, this study has several strengths that enhance the robustness and significance of this findings. Firstly, by conducting preoperative, intraoperative, and postoperative measurements of vital signs, including systolic and diastolic blood pressure, pulse rate, saturation, fever, and pupil diameter, we captured a comprehensive picture of physiological responses during third molar extraction surgery. The inclusion of multiple time points enabled us to observe dynamic changes in vital signs throughout the surgical procedure, providing valuable insights into the temporal relationship between anxiety and physiological parameters. Moreover, the study’s focus on a specific surgical procedure, third molar extraction, allowed for a more targeted investigation of anxiety-inducing factors and their impact on vital signs in a clinical context. Overall, these strengths underscore the significance of this study in advancing understanding of the complex interplay between psychological factors and physiological responses during dental procedures.

## Conclusions

Fear and anxiety are important issues to address to make patients more comfortable for surgery. Dental surgery for impacted third molars is a procedure that increases anxiety levels in patients. Therefore, methods to reduce such anxiety should be explored, and broader studies on anxiety should be conducted. In conclusion, the present study helps elucidate the relationship between dental anxiety and vital signs during oral surgery. However, further research with a larger and more diverse sample is recommended to validate and generalise the findings of this study. In addition, it would be valuable to investigate the long-term impact of dental anxiety on vital signs and patient outcomes for both clinical practice and future research in this field.

## Data Availability

The datasets used and analysed during the current study are available from the corresponding author on reasonable request.
